# Placenta Powder-Infused Thiol-Ene PEG Hydrogels as Potential Tissue Engineering Scaffolds

**DOI:** 10.1021/acs.biomac.2c01355

**Published:** 2023-03-21

**Authors:** Yanmiao Fan, Mads Lüchow, Adel Badria, Daniel J. Hutchinson, Michael Malkoch

**Affiliations:** Division of Coating Technology, Department of Fibre and Polymer Technology, KTH Royal Institute of Technology, Teknikringen 56-58, 10044 Stockholm, Sweden

## Abstract

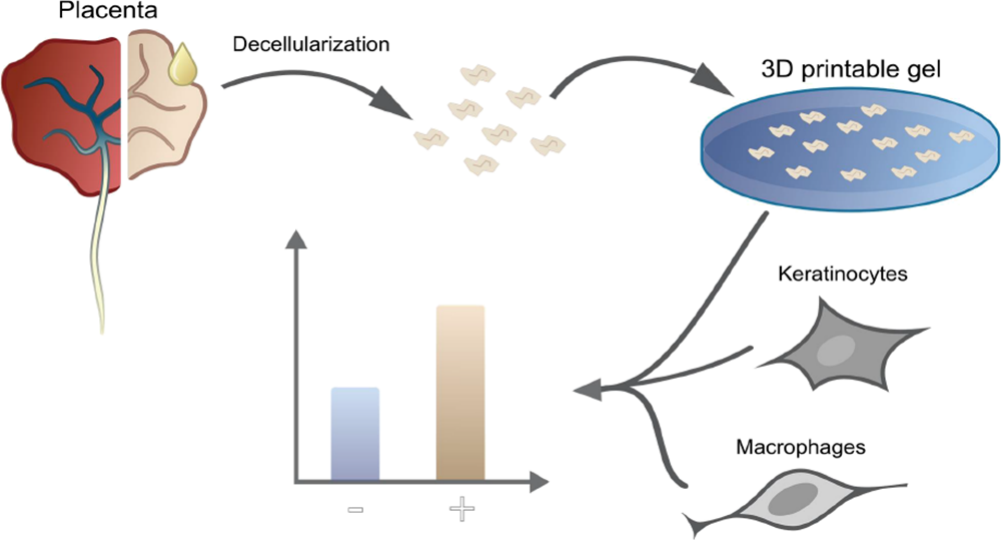

Human placenta is a source of extracellular matrix for tissue engineering. In this study, placenta powder (PP), made from decellularized human placenta, was physically incorporated into synthetic poly(ethylene glycol) (PEG)-based hydrogels via UV-initiated thiol-ene coupling (TEC). The PP-incorporated PEG hydrogels (MoDPEG+) showed tunable storage moduli ranging from 1080 ± 290 to 51,400 ± 200 Pa. The addition of PP (1, 4, or 8 wt %) within the PEG hydrogels increased the storage moduli, with the 8 wt % PP hydrogels showing the highest storage moduli. PP reduced the swelling ratios compared with the pristine hydrogels (MoDPEG). All hydrogels showed good biocompatibility in vitro toward human skin cells and murine macrophages, with cell viability above 91%. Importantly, cells could adhere and proliferate on MoDPEG+ hydrogels due to the bioactive PP, while MoDPEG hydrogels were bio-inert as cells moved away from the hydrogel or were distributed in a large cluster on the hydrogel surface. To showcase their potential use in application-driven research, the MoDPEG+ hydrogels were straightforwardly (i) 3D printed using the SLA technique and (ii) produced via high-energy visible light (HEV-TEC) to populate damaged soft-tissue or bone cavities. Taking advantage of the bioactivity of PP and the tunable physicochemical properties of the synthetic PEG hydrogels, the presented MoDPEG+ hydrogels show great promise for tissue regeneration.

## 1 Introduction

Surgical grafting remains the primary treatment for patients suffering from severe tissue damage that requires replacement of the damaged tissue. There are, however, significant issues with grafting as a treatment plan, including donor site morbidity, limited availability, rejection, and disease transfer, depending on whether auto-, allo-, or xenografts are used.^1−3^ Tissue engineering, as an alternative to treat missing or damaged tissue, has steadily garnered interest since the popularization of the field by Green and Gallico in the late 1970s and early 1980s.^4−7^ In an attempt to ensure a high degree of similarity between the fabricated matrix and the tissue it aims to emulate, many have used mammalian-derived donor tissues as a base for tissue engineering scaffolds.^8−15^ These often come in the form of extracted collagen or intact, decellularized ECM from bovine or porcine sources^8,13^ or from human cadavers.^14,15^ However, mammalian-derived materials for tissue regeneration commonly require a sacrificial donor, potentially leading to the unnecessary slaughter of farm animals and limited availability. As such, renewable, expendable sources of mammalian tissue are needed. Mammalian placenta is a suitable candidate, as it provides a source of tissue that is often discarded following delivery and thus requires minimal sacrifice. Indeed, the human placenta has been successfully employed as an ECM substitute and scaffold in tissue engineering.^16−18^ Decellularized human placenta could be formulated into hydrogels to induce a higher density of blood vessels in vivo compared with a collagen type I hydrogel.^19^ Decellularized human placenta amniotic membrane (HAM) collagen matrix was also used to form functional hydrogels together with methacrylated gelatin,^20^ and hyaluronic acid^21^ for tissue engineering. Choi et al. made thin sheets of decellularized, disrupted human placenta and, upon wound application on a murine model, saw evidence of keratinocyte and epithelial cell migration being promoted by the device.^16^ However, like most natural polymer-based hydrogel scaffolds, decellularized placenta alone lacks flexibility in its mechanical properties and they are less stable due to enzyme degradation. Disrupted or pulverized placenta is limited by the shapes that can be molded and cast and manipulating the mechanical strength of the tissue would likely entail disrupting the composition of the structural proteins which also provide the cells with important signaling cues. A potential solution to the shortcomings of human placenta-based hydrogel scaffolds is to combine the bioactivity of human placenta and the tunable physicochemical properties of synthetic hydrogels. PEG is a hydrophilic polymer that has been approved by the FDA, and synthetic PEG hydrogels have shown great promise in soft-tissue engineering due to their good biocompatibility, adjustable mechanical properties, and degradability.^22^ However, since most synthetic hydrogels are bio-inert, hydrogel modification with bioactive moieties or the addition of active agents is important to provide the synthetic hydrogels with good cell adhesion and an optimized environment for cell proliferation.^23^ PEG-based hydrogels have been modified with functional peptides or RGD to provide the hydrogel scaffolds with good cell adhesion properties.^22,24,25^ The combination of bioactive placenta powder and synthetic PEG hydrogels is promising as a tissue engineering scaffold. In addition, 3D printing technology is of rising interest in the tissue engineering community due to its potential to create bioactive scaffolds with a complex topology that complies with the unique and personalized character of wound site profiles.^26−29^ Multiple 3D printing technologies exist, with fused deposition modeling (FDM), stereolithography (SLA), and selective laser sintering (SLS) being some of the most common; however, some are better suited for tissue engineering than others, and there are advantages and disadvantages to each technology.^26,29−31^ For tissue engineering specifically, a newer technique called bioprinting, where a viscoelastic biomaterial is deposited onto a built surface layer-by-layer through a thin nozzle, is the most popular.^31,32^ The popularity of the method stems from the flexibility in the materials that can be used during printing, which allows for the build-up of 3D structures from multiple different materials and even direct deposition of cell-laden hydrogels.^27,31−33^ The limiting factor of bioprinting, however, is the print resolution, which, similarly to FDM printing, is dictated by the nozzle size, which is rarely smaller than 200 μm, and the flow properties of the printing material.^26,28,31,34^ Higher-resolution printing can be achieved using methods such as SLA and laser-assisted bioprinting (LAB), which employ light to cure specific parts of a photocurable material, as the resolution of these methods is dictated by the size of the laser.^26,31^

**Figure 1:**
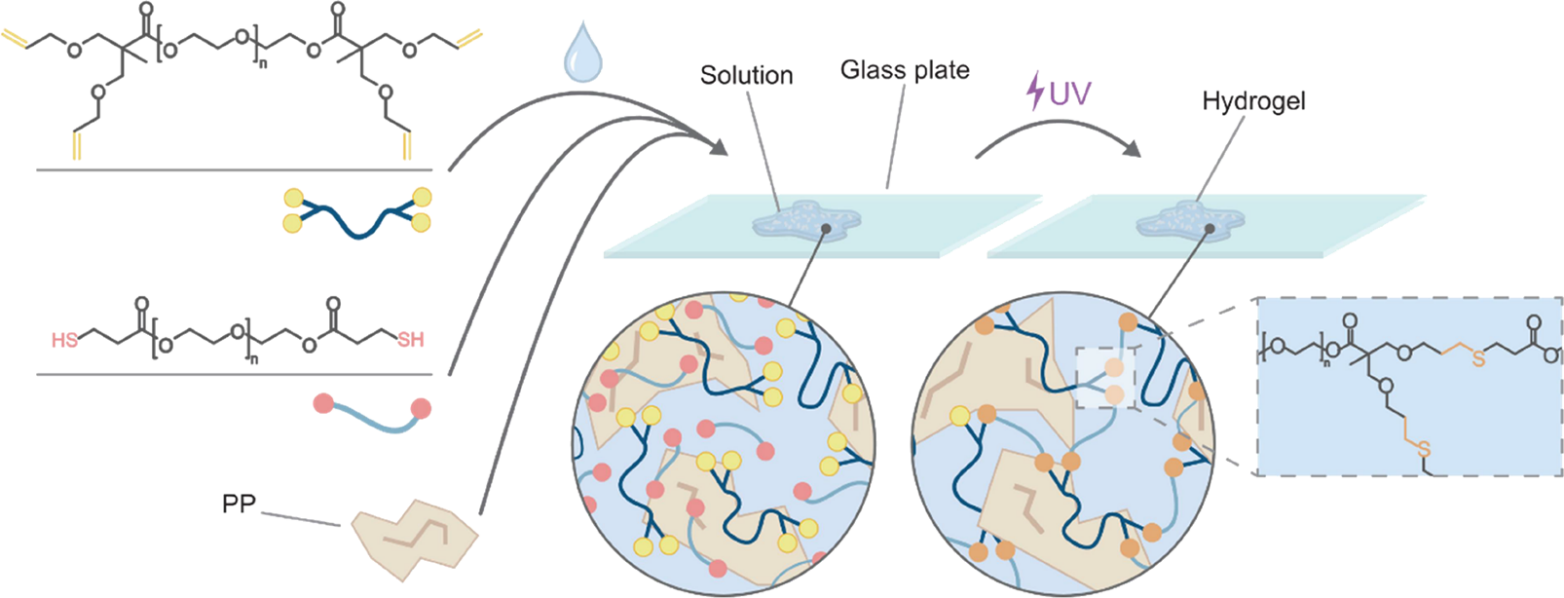
Schematic diagram describing the formation of MoDPEG+ hydrogels via thiol-ene reaction.

The work presented in this paper explores the potential of decellularized placenta powder (PP) encapsulated bioactive PEG hydrogels (MoDPEG+) and pristine PEG hydrogels (MoDPEG) as potential scaffolds for tissue engineering. Fast and efficient formation of these MoDPEG and MoDPEG+ networks is ensured through thiol-ene click cross-linking reactions (Figure 1).^35^ These PEG hydrogels, formed from PEG functionalized with allylated bis(hydroxymethyl)propionic acid and thiol terminated 3-mercaptopropionic acid, possess a range of features that are important for tissue engineering, such as good biocompatibility, adjustable swellability, degradability, and tunable stiffness comparable to damaged tissue.^36,37^ Importantly, the introduction of decellularized PP into the hydrogel system provides a bioactive segment for cell adhesion and proliferation in the bio-inert PEG hydrogels. The impact of incorporated PP on the mechanical properties and swelling of the hydrogels was investigated, along with in vitro cytotoxic evaluation of the materials using murine macrophages (Raw 264.7), human keratinocytes (Hacat) and human dermal fibroblasts (HDF). Additionally, the 3D printability of the PP-containing hydrogels, which was made possible by the photoinitiated thiol-ene coupling, was demonstrated using SLA technology.

## 2 Experimental Section

### 2.1 Materials

Placentae were obtained from Karolinska University Hospital, Stockholm, Sweden, with consent from donors. Information about the donors is shown in Table S1. Allyl bromide (97%), *N*,*N*′-dicyclohexylcarbodiimide (DCC, 99%), 4-(dimethylamino)pyridine (DMAP, 99%), DNase I from bovine pancreas, Eosin Y (99%), lithium phenyl-2,4,6-trimethylbenzoylphosphinate (LAP), 3-mercaptopropanoic acid (99%), phenolphthalein, pyridine, sodium dodecyl sulfate (SDS, 99%), and sodium hydroxide (NaOH, 98%) were purchased from Sigma-Aldrich Sweden AB, Stockholm, Sweden. 2,2-Bis(hydroxymethyl)propionic acid (bis-MPA) was obtained from Perstorp AB, Malmö, Sweden. Chloroform-d (CDCl_3_), dichloromethane (DCM), hydrochloric acid (HCl 37%), peracetic acid (PAA, 38%), polyethyleneimine (PEI, 10 kDa, branched), and p-toluenesulfonic acid (pTSA, 98%) were purchased from VWR International AB, Spånga, Sweden. All reagents were used as received unless otherwise stated. PEG of Mw 2, 6, 10, and 20 kDa was purchased from Sigma-Aldrich Sweden AB, Stockholm, Sweden, and dried with toluene before use. QIAamp DNA mini kit was purchased from Qiagen AB, Sollentuna, Sweden. HDF, human keratinocytes (Hacat), and murine macrophage (Raw 264.7) cells were purchased from the American tissue culture collection in Manassas, Virginia, USA.

### 2.2 Instruments

Rheology measurements were performed on a Discovery series Hybrid Rheometer-2 (TA Instruments). Spectrofluorometry was conducted on an Infinite M200 Pro plate reader (Tecan Group Ltd., Switzerland). Optical density was measured on a mySPEC microvolume spectrophotometer (VWR International LLC). Placenta was ground using a PM 400 planetary ball mill (Retsch GmbH, Germany).

### 2.3 Placenta Decellularization and Fragmentation

A modified version of the protocol presented by Choi et al.^16^ was used in the decellularization of placenta. The protocol was applied to both frozen and fresh placenta. In the case where nonfrozen placenta was prepared, the tissue was put on ice and prepared no more than 24 h after harvest. First, the tissue was washed several times with deionized (DI) water to remove blood components before subsequent homogenization. In the case where frozen placenta was prepared, the tissue was thawed and gradually washed in DI water. Once washed, tissue and DI water were added to a VitaMix blender in a 1:1 (w/w) ratio. The tissue was blended on high in 30 second intervals for a total of 5 min, letting the appliance cool off in-between intervals. The mixture was then centrifuged at 3000 *g* for 5 min and the supernatant was discarded. The tissue was resuspended in DI water, centrifuged at 3000 *g* for 5 min, and the supernatant was discarded. This procedure was repeated until the Kastle–Meyer’s test no longer showed traces of blood in the supernatant, which required at least 5 cycles. The disrupted tissue was resuspended in 0.5% SDS to make a 1:1 (v/v) mixture, which was left on a shaking table at room temperature for 30 min. The mixture was then centrifuged and the supernatant was discarded (3000 *g*, 5 min). The placenta was washed with DI water at room temperature 6 times with a minimum of 1 h between washes. The tissue was then treated with 0.2% DNase for 10 min at 37 °C. After centrifugation and removal of the supernatant (3000 *g*, 5 min), the tissue was washed with DI water 3 times. Then, 0.1% PAA was added to make a 1:1 (v/v) mixture, which was left on a shaking table for 30 min at room temperature. The mixture was centrifuged (3000 *g*, 5 min) and the supernatant was removed in a sterile environment. The tissue was washed several times under sterile conditions for 2 days, and subsequently resuspended in DI water (1:1 (v/v)) and lyophilized for 48 h. The lyophilized placenta was first ground in liquid nitrogen using a mortar and pestle to reduce the size of the largest pieces of decellularized tissue. Following manual grinding, the material was ground on a planetary ball mill, using stainless steel grinding jars and balls (50 mL, 1 cm diameter, respectively). The tissue was ground for a total of 1 h in 5 min intervals with 10 min breaks between intervals to avoid overheating and potential denaturation of ECM proteins. The pulverized placenta powder particles were then separated using laboratory sieves, and particles measuring smaller than 125 μm, which comprised 92 wt% of the particles, were collected and used in hydrogel fabrication.

### 2.4 Characterization of Decellularized PP

#### 2.4.1 Kastle–Meyer’s Test

A Kastle–Meyer reagent was made by dissolving NaOH in 70% ethanol (5 mL) to create a 25% NaOH solution. Phenolphthalein (0.05 g) was then dissolved in the solution followed by the addition of zinc (0.05 g), which was dissolved through gentle boiling. During the dissolution of zinc, the solution turned from bright pink to pale yellow. Once cooled, the solution was diluted to 50 mL with 70% ethanol. The Kastle–Meyer’s test was performed by letting the supernatant soak into a piece of filter paper, which was then left to dry for 30–60 s. A drop of Kastle–Meyer reagent was then deposited on the filter paper, followed by a drop of hydrogen peroxide and the color of the filter paper was monitored. A pink coloration upon contact with the hydrogen peroxide would indicate that blood was still present and the tissue needed to be washed once more; otherwise, it would be subjected to the next step in the decellularization process as described above.

### 2.5 SDS Concentration

SDS detection was performed by a turn-on fluorescent sensor reaction presented by Wen et al.^38^ Briefly, HAc-NaAc buffer solution (pH 4.0) along with 0.1 mM stock solutions of PEI and Eosin Y, respectively, were prepared and a supernatant sample from the wash-step following SDS treatment of the placenta (see decellularization procedure above) was collected. The buffer (311 μL), PEI solution (5 μL), Eosin Y (4 μL), and supernatant (80 μL) were transferred to a single well of a 96-well plate and mixed, creating a mixture containing 1 μM Eosin Y, 0.8 μM PEI, and an unknown amount of SDS. Fluorescence intensity, *F*, was then measured by a plate reader at excitation and emission wavelengths of 512 and 541 nm, respectively, and plotted against the intensity of a blank reagent, *F*_0_, which was prepared in a similar fashion, albeit with DI water (80 μL) rather than supernatant. A cycle of washing the placenta tissue followed by SDS detection was then repeated until the relative fluorescence, *F*/*F*_0_, had reached a steady state indicating complete removal of SDS from the washed tissue.

### 2.6 DNA Concentration

Tissue samples were prepared for DNA concentration measurement at the beginning and end of the decellularization process using a QIAamp DNA mini kit. A tissue sample was taken and then separated into three small samples of approximately 25 μg for the purpose of statistics: exact weights were recorded for each subsample. Each sample was treated with a range of buffers and proteinases in accordance with the manufacturer’s guidelines and the absorbance was measured at 260 nm to determine nucleic acid concentration.

### 2.7 Material Synthesis

Bis(allyl propionic acid) (BAPA), BAPA anhydride, PEG-BAPA, and 2k-PEG bis(3-sulfanylpropanoate) (PEG-SH) were synthesized according to methods presented in previous studies by our group.^35,37^

### 2.8 Hydrogel Formation

MoDPEG hydrogels were fabricated as described in previous studies.^37^ A representative 1 mL MoDPEG+ hydrogel containing 20 wt % PEG species and 4 wt % PP was produced as follows: PEG-BAPA (141 mg, 10 kDa), PEG-SH (59 mg, 2 kDa), and PP (40 mg) were added to an amber vial and mixed thoroughly on a vortex mixer. The relation between PEG-BAPA and PEG-SH created a 1:1 allyl-to-thiol ratio. DI water (640 μL) was added, and the mixture was vortex mixed to completely dissolve the PEG species. Once dissolved, LAP solution (120 μL, 20 mg·mL^–1^ in DI water) was added resulting in a 1.2 wt % concentration of LAP. The solution was mixed and left in a dark environment to de-foam. Once devoid of bubbles, the solution was drop casted or injected into desired shapes and cured by exposure to a 6 J cm^–2^ dose of UV light, resulting in a transparent, elastic hydrogel containing opaque placenta particles. The formulation was adjusted accordingly for gels containing other amounts of PEG and PP.

### 2.9 FTIR

A Perkin-Elmer Spectrum 100 FTIR spectrometer with ATR attachment was used to analyze the PP and the hydrogels. The samples were freeze-dried and hydrogels were fragmented before the measurement. Each spectrum was recorded with a 4 cm^–1^ resolution between 600 and 4000 cm^–1^ as an average of 32 scans. The data were processed using the Perkin-Elmer software spectrum.

### 2.10 Scanning Electron Microscopy (SEM)

SEM was used to characterize the hydrogels. Briefly, the hydrogels were freeze-dried and fractured with a sharp razor, then coated with Pt in a 208HR high-resolution sputter coater (Cressington, Watford, UK). SEM analysis was conducted using S-4800 field emission scanning electron microscope (Hitachi, Tokyo, Japan).

### 2.11 Rheology

Rheology measurements were conducted on swelled gels at 37 °C using a Discovery Hybrid Rheometer 2 from TA Instruments. Measurements were performed on triplicate samples as amplitude sweeps at a 1 Hz frequency ranging from 0.1 to 200% strain using a 25 mm parallel plate geometry. An initial sample of each set of triplicates was compressed to a thickness of 500–850 μm and the loss and storage moduli were measured at this distance. The axial force was recorded and used as a target for the remaining samples in that set of triplicates to ensure consistency within sets of measurements.

### 2.12 Swelling

The swelling ratios of the hydrogels were measured in DI water at 37 °C. After UV curing, the hydrogels were freeze-dried, weighed and immersed in DI water and transferred to an incubator at 37 °C. The hydrogels were weighed at different time intervals until their weight remained constant, indicating that the equilibrium had been reached. The experiment was done in triplicate and the equilibrium swelling ratio (ESR) was calculated using eq 1, where *W*_s_ is the weight of swollen hydrogel and *W*_d_ is the weight of the hydrogel after freeze drying:
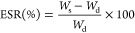
1

### 2.13 Cytotoxicity Screening

Hydrogels containing 20 wt % PEG (10 kDa) and 4 and 8 wt % PP, respectively, were produced for cytotoxicity screening. The hydrogels were sterilized under UV for 30 min and immersed in sterilized DI water for 6 h for swelling at 4 °C to limit unwanted degradation. The swollen hydrogels were subsequently transferred to Dulbecco’s Modified Eagle Medium (DMEM) containing 10% fetal bovine serum (3 mL) and incubated for 24 h at 37 °C, leading to a hydrogel dry weight-to-medium ratio of 10 mg·mL^–1^. HDFs, Hacat and Raw 264.7 cells, were seeded in 96-well plates with cell concentration of 10,000 cells per well. One hundred microliters of the hydrogel-laden medium were added to these wells and the 96-well plates were transferred to an incubator and incubated for 72 h at 37 °C. After 72 h of incubation, 10 μL Alamar Blue (31:5 dilution) was added to each well. Cells were then incubated for an additional 4 h at 37 °C and the fluorescence intensity was subsequently measured at ex/em 560/590 nm.

### 2.14 Cell Growth on the Hydrogel Scaffolds

A thin film hydrogel (20 μL) was formed in a 1 mL syringe, and the hydrogels were immersed in PBS for swelling for 6 h at 4 °C, subsequently, the swollen hydrogels were transferred into 48 well plates and 400 μL cell solution (1 × 10^5^ cells/mL) was added to each well. Cell adhesion and proliferation were observed under the microscope. The experiment was done in triplicate. Since the hydrogels did not occupy the whole bottom of the well, some cells would land on the hydrogels while others would land at the bottom: this aided in the observation of cell behavior and ensured that cells could move freely from the hydrogels.

### 2.15 3D Printing

3D-printed hydrogels were printed on a commercial Peopoly Moai 130 SLA 3D printer with custom small vat and platform attachments to decrease material waste. For better print adhesion, a glass substrate was glued to the surface of the metal build platform. 3D models were generated using AutoCAD, and STL-files were sliced using Cura 4.7 software at 100 μm at 1 mm·s^–1^ speed. Hydrogel solutions with a dry weight content of 50 wt % and including 6K-PEG-BAPA (6K50%), were formulated as described above, albeit with 0.83 wt % LAP used and the addition of a nontoxic water-soluble photoabsorber (Tartrazine) to control curing depth. Penetration depth (*D*_P_) was measured using a digital microscope (Dino lite Premier) and critical curing energy (*E*_c_) was obtained as the *x*-axis intersection of a linear fit of Jacob’s working curve (Figure S6). The hydrogel in the shape of the KTH logo was intentionally overcured to form a singular hydrogel from the separate elements of the logo, rather than adding a raft in order to save on material.

### 2.16 Statistical Analysis

Statistics were performed using two-tailed, independent Student’s T-Test using Microsoft Excel. Significant statistical difference in figures is indicated by asterisks where appropriate. Values are expressed as mean ± standard deviation. For all statistical analyses, *p* < 0.05 was considered significant.

## 3 Results and Discussion

### 3.1 Placenta Decellularization and Characterization

**Figure 2:**
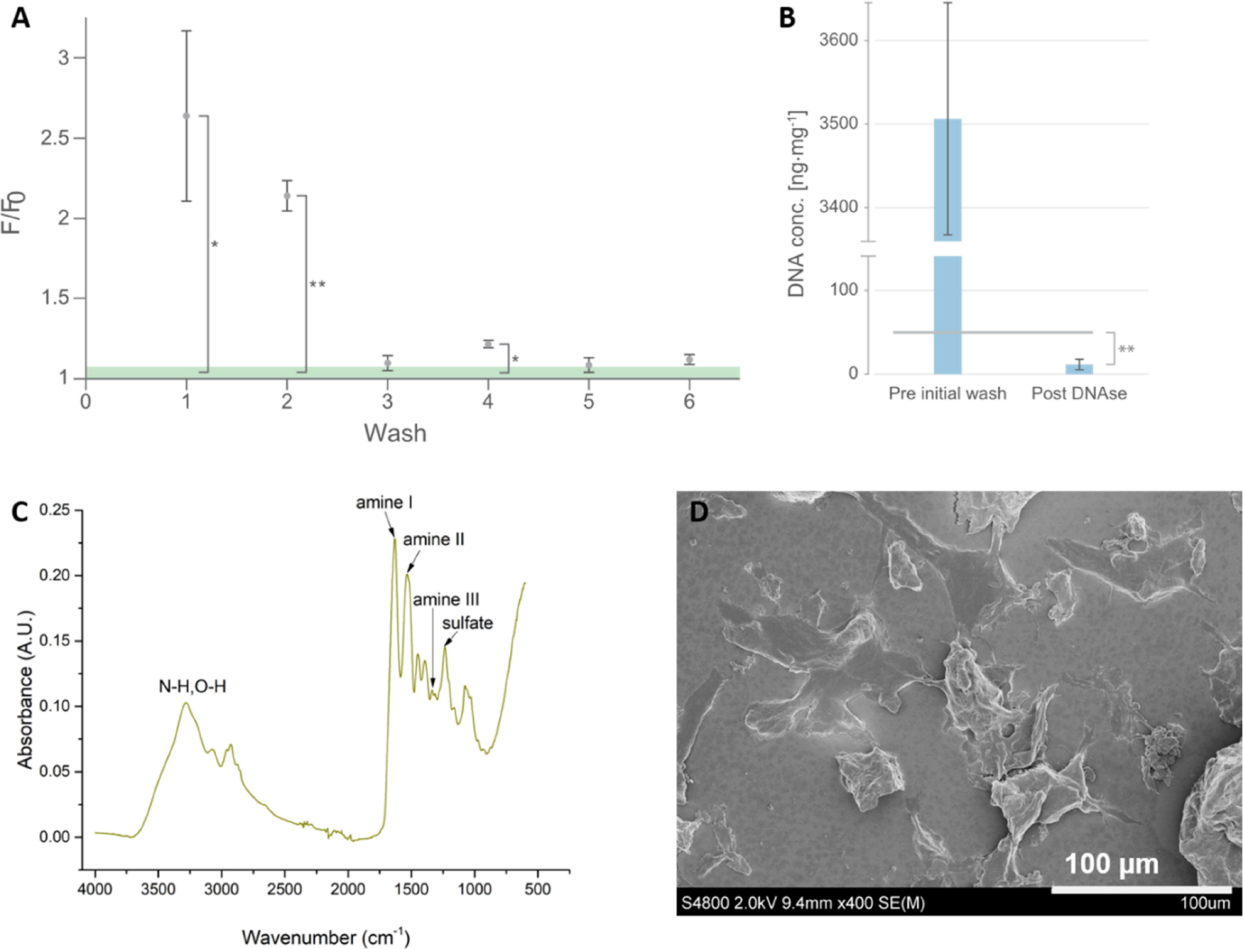
(A) Effect of washing on the remaining SDS in placenta tissue after SDS treatment as measured by the relative fluorescence, *F*/*F*_0_, from the dye Eosin Y. Light green area indicates standard deviation of the baseline measurement (blank); (B) DNA concentration in ng·mg^–1^ tissue before the first decellularization step and after the last step of decellularization. Gray bar indicates the allowed DNA concentration limit as proposed by Crapo et al.^39^ (C) FTIR analysis of decellularized PP. (D) SEM of the decellularized PP. Error bars show standard deviation (*n* = 3); asterisks show *p*-values < 0.05; double asterisks show *p*-values < 0.005, *n* = 3.

Placenta decellularization was conducted based on previously published papers with some modifications.^16^ The decellularized placenta was lyophilized into a dry, coarse placenta powder and separated into different sizes using a sieve. SDS was used to remove cellular components and debris from the placenta; however, it was then necessary to ensure the complete removal of SDS through repeated washes with deionized water, since trace amounts of SDS in decellularized tissue samples can cause undesired side effects, such as interference with the activity of the DNase that was used in the following step, or cause cytotoxicity effects. We detected the effect of different wash cycles on the removal of SDS using a turn-on fluorescence reaction originally presented by Wen et al.^38^ In this reaction, fluorescence from the dye Eosin Y was used to indicate the presence of SDS, as in the absence of SDS the fluorescence is quenched through the association of Eosin Y and polyethyleneimine (PEI). Based on the relative fluorescence, *F*/*F*_0_, the supernatant was seemingly devoid of SDS after three washes (Figure 2A). However, the supernatant from the fourth wash indicated a statistically significant amount of SDS relative to the blank baseline, *F*_0_ (*p* < 0.05), which could have been caused by tissue remnants present in the supernatant. In order to avoid this, the tissue was washed thoroughly more than 6 times after SDS treatment. DNA content in the placenta was also measured before and after the decellularization procedure. As shown in Figure 2B, a massive reduction in DNA content in the tissue was observed following decellularization, from 3506 ± 139 to 12 ± 7 ng·mg^–1^ tissue, indicating the successful removal of cellular components as they were well below the threshold for proper decellularization of <50 ng DNA per mg tissue as defined by Crapo et al.^39^

Fourier-transform infrared spectroscopy (FTIR) was used to characterize the decellularized PP. As shown in Figure 2C, PP showed absorption peaks at 1631 (amide I), 1532 (amide II), and 1336 cm^–1^ (amide III). These three peaks indicated the existence of collagen in the decellularized PP.^19^ The FTIR spectrum also indicated that sulfate groups (around 1235 cm^–1^ for the S=O stretch of R-SO_3_^–1^) and sugar residues (C–C–O and C–O–C stretches, from 1000 to 1200 cm^–1^) were present in the PP. SEM was used to characterize the morphology of decellularized PP (Figure 2D). Decellularized PP showed irregular shapes, and the size of the PP particles was smaller than 125 μm due to the use of a laboratory sieve to separate the PP after decellularization.

### 3.2 Hydrogel Formation and Characterizations

**Figure 3:**
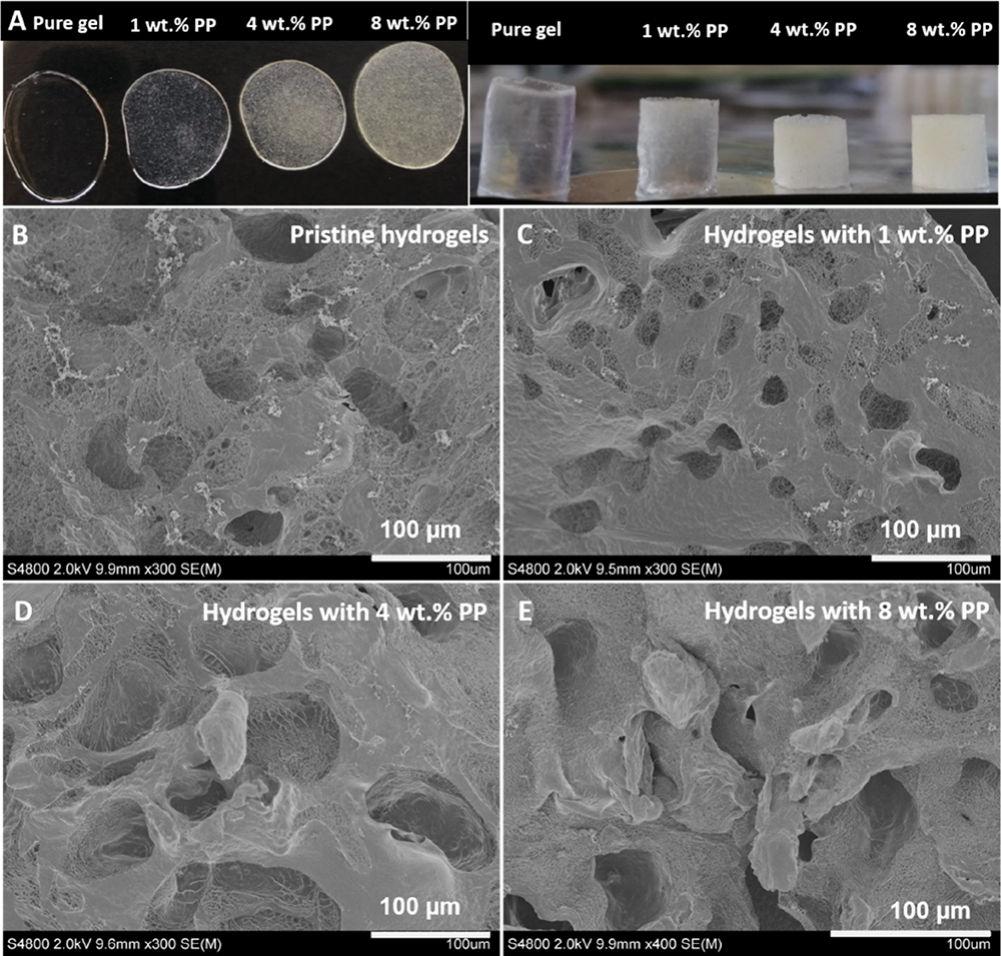
(A) Thin film and cylindrical MoDPEG and MoDPEG+ hydrogels (formed with 10KPEG, 20 wt % PEG) with different percentages of PP. (B–E) SEM images showing the porous structures of MoDPEG hydrogel and MoDPEG+ (10KPEG) hydrogels with PP content of 1, 4, and 8 wt %.

MoDPEG and MoDPEG+ hydrogels with different PEG lengths and dry weight contents were formed via facile thiol-ene reactions.^37^ In order to prepare MoDPEG+ hydrogels, PP was added to the precursor solution, which was then well mixed before being cured with UV light (Figure 1). Different percentages of PP were incorporated into the hydrogels to find the most bioactive PP and PEG hydrogel combination for supporting cell adhesion and growth. PP particles had the tendency to sediment if the hydrogel precursor solution was left stationary; in this study, PP particles smaller than 125 μm were used to form MoDPEG+ hydrogels mainly because they could be dispersed in the precursor mixture homogeneously, and it took a longer time for smaller PP to sediment to the bottom. The sedemented PP could be easily redispersed through light agitation of the mixture, resulting in placenta particles spread evenly throughout the hydrogels (Figure 3A). The pristine MoDPEG hydrogels were transparent due to the hydrophilic PEG; however, their transparency decreased with increasing concentration of the opaque PP, which was observed with the naked eye (Figure 3A) or with a microscope (Figure S1). SEM images showed that all the hydrogels had a porous structure (Figure 3B–E). Compared with the pristine MoDPEG hydrogel, MoDPEG+ hydrogels showed less fiber structures, probably due to the interactions between PEG and PP, and the compact structure probably contributed to the enhancement of the mechanical properties of the MoDPEG+ hydrogels. In addition, the porous structure was a desired outcome as a highly porous hydrogel structure is beneficial for tissue engineering.^40^

**Figure 4:**
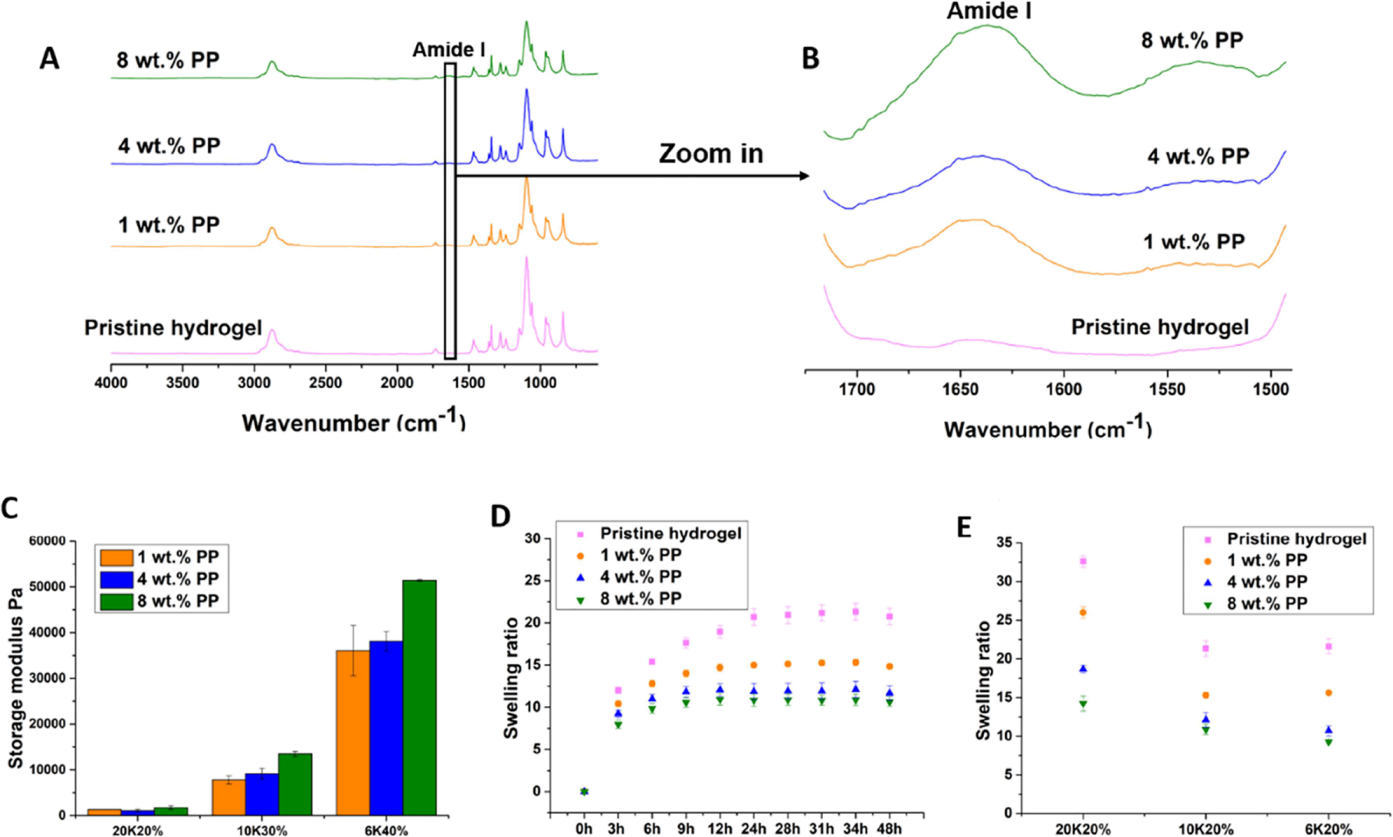
(A) FTIR analysis of pristine MoDPEG hydrogels and MoDPEG+ hydrogels. (B) Zoomed-in image of FTIR spectra showing the amide I peak at 1631 cm^–1^ in MoDPEG+ hydrogels. (C) Mechanical properties of MoDPEG+ hydrogels. (D) Swelling ratios of the hydrogels formed with 10KPEG in DI water at 37 °C at different time intervals. (E) Swelling ratios of MoDPEG and MoDPEG+ hydrogels formed with 20KPEG, 10KPEG, and 6KPEG. Error bars show standard deviation. Data are shown as mean value ± SD (*n* = 3).

FTIR (Figure 4A,B) showed the representative collagen peaks of amide I at 1631 cm^–1^ in all MoDPEG+ hydrogels due to the presence of PP, while the collagen peaks were absent in the pristine MoDPEG hydrogels. The effect of PP on the mechanical properties of the hydrogels was also investigated by adjusting the PP contents, dry weight contents (20, 30, and 40 wt %) and PEG (6KPEG, 10KPEG, and 20KPEG) length. The abbreviated names of the hydrogels in Figure 4C were based on the dry weight content and PEG length; for example, hydrogels containing 20 wt % dry weight content and 20K-PEG-BAPA were abbreviated as 20K20%. MoDPEG+ hydrogels showed a wide range of storage moduli ranging from 1084 ± 293 to 51,411 ± 199 Pa (Figure 4C), which matched the moduli of several soft tissues such as endothelial, skeletal muscle, and uterine smooth muscle tissue.^41−43^ MoDPEG+ hydrogels showed a general increase in elastic modulus with increasing PP content from 1 to 8 wt % (Figure 4C). The increase in modulus associated with the addition of PP was consistent with the addition of a physically entrapped filler in composite materials. The PP appeared to enhance the compactness of the MoDPEG+ hydrogels shown in SEM images, thus reducing the flexibility of the hydrogel and increasing its modulus. Moreover, PP is hydrophobic and the encapsulation of PP reduced the swelling ratios of the hydrogels (Figure 4E), which probably also contributed to the increased storage moduli. This meant that in addition to varying the length of the PEG-BAPA moiety and the PEG wt %, the modulus of the MoDPEG+ hydrogels can be reliably controlled by adjusting the concentration of PP.

The effects of PP on swelling ratios of the hydrogels were also explored. Figures 4D and S2 show the swelling of the pristine MoDPEG and MoDPEG+ hydrogels (20 wt % dry weight) formed with 10KPEG, 6KPEG, and 20KPEG. The MoDPEG+ hydrogels absorbed the majority of their water intake within 3 h, with the 10KPEG and 6KPEG gels reaching equilibrium after 9 or 12 h and the 20KPEG gel after 24 h. The pristine MoDPEG hydrogels showed higher swelling ratios and longer times to reach equilibrium compared with their corresponding MoDPEG+ hydrogels. The increase of PP content decreased the swelling ratios (Figure 4E), with the lowest swelling ratio of 9.3 ± 0.22 achieved by hydrogels formed with 6KPEG hydrogels and 8 wt % PP content, while the highest swelling ratio of 32.6 was achieved by the pristine MoDPEG hydrogel formed with 20KPEG (Figure 4E). These results indicated that the encapsulation of PP reduced the swelling of the PEG hydrogels; which was probably due to the PP consisting mainly of hydrophobic collagen.

### 3.3 In Vitro Cytotoxicity

**Figure 5:**
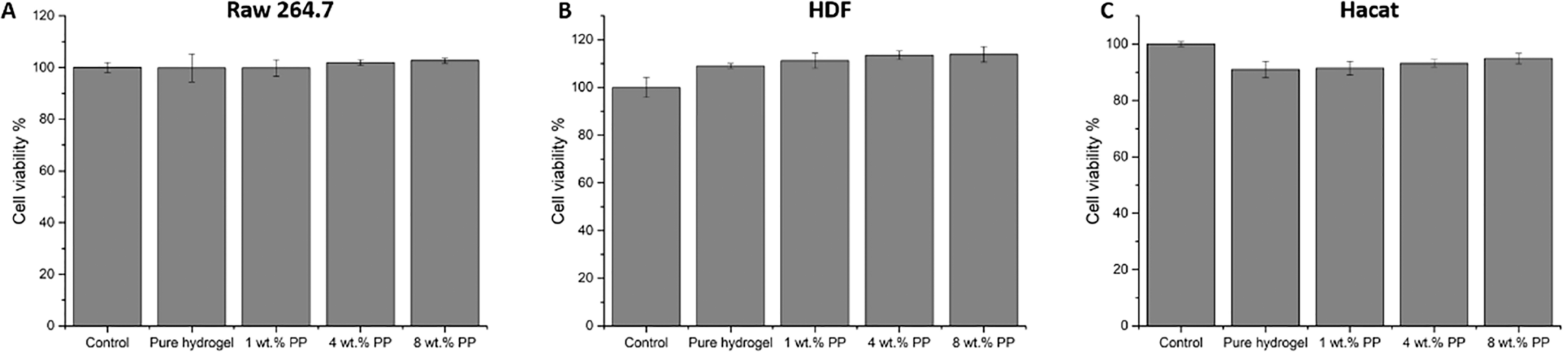
Cytotoxicity evaluation of MoDPEG and MoDPEG+ hydrogels on (A) Raw 264.7 cells. (B) DHF cells and (C) Hacat cells determined by AlamarBlue assay after incubation for 24 h. All data are shown as mean value ± SD (*n* = 6).

As potential tissue engineering materials for skin wound healing, good biocompatibility is one of the most basic and important requirements. Hydrogels formed with 10KPEG and dry weight percentage of 20 wt % have shown good biocompatibility and mechanical strength comparable to human skin in our previous study;^37^ therefore, 10K20% hydrogels with and without PP were used for the cytotoxicity study and cell adhesion and proliferation study. The biocompatibility of MoDPEG and MoDPEG+ hydrogels was evaluated in vitro using Raw 264.7, HDF, and Hacat cells. As shown in Figure 5, neither the pristine MoDPEG hydrogels nor any of the MoDPEG+ hydrogels with added PP showed any toxicity toward Raw 264.7 (Figure 5A, cell viability above 99.7%), HDF (Figure 5B, cell viability above 100%), or Hacat cells (Figure 5C, cell viability above 90.9%). These findings align with the literature, where decellularized placenta showed good biocompatibility and potential as tissue engineering materials.^16,19^ Notably, MoDPEG and MoDPEG+ hydrogels seemed to enhance the growth and cell viability of DHF cells, indicated by cell viability ranging from 109 to 113.8% compared with cells in the control group. Our results suggested that the addition of PP within the synthetic PEG hydrogels showed good biocompatibility and great promise as tissue regenerative scaffolds.

### 3.4 Cell Adhesion and Proliferation on the MoDPEG and MoDPEG+ Hydrogels

**Figure 6:**
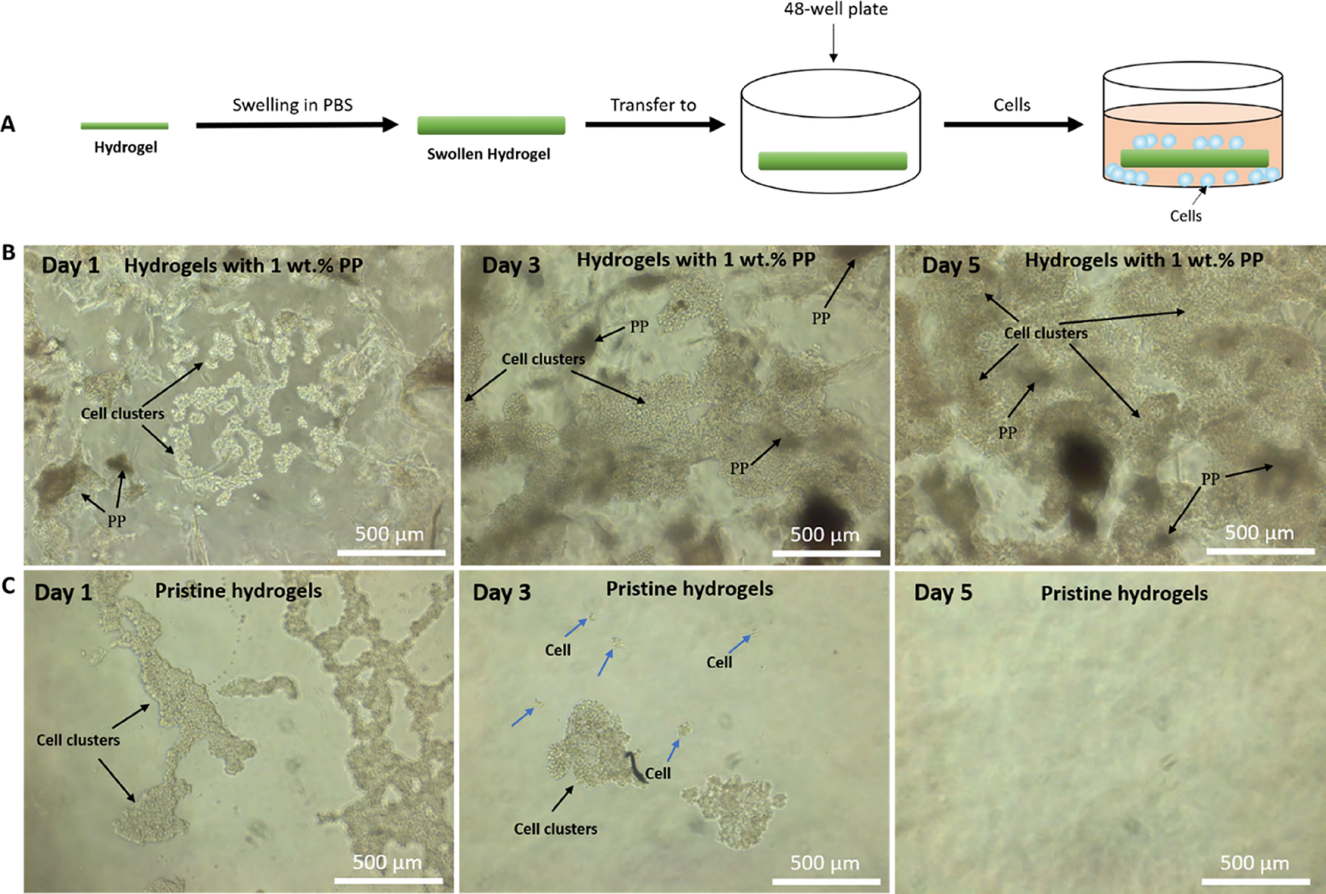
(A) Schematic illustration of cell loading on the hydrogels. (B) Raw 264.7 cell adhesion and proliferation on MoDPEG+ hydrogels (dark and opaque clusters were PP). (C) Raw 264.7 cell adhesion and proliferation on pristine hydrogels. The blue arrows indicate cells that have de-attached from the large cell clusters and have moved away from the hydrogel surface.

Previous studies have shown that PEG hydrogels are bio-inert and cannot support the adhesion and growth of cells; therefore, bioactive modification is of great importance for PEG hydrogels.^23^ PEG hydrogels functionalized with RGD peptide can support human mesenchymal stem cells (hMSC) adhesion and growth, while bio-inert PEG hydrogels cannot support the adhesion.^24^ The ability of the MoDPEG+ hydrogels to adhere and proliferate Raw 264.7 and Hacat cells was investigated to determine if PP can encourage cell adhesion and proliferation. As shown in Figure 6A, a thin film of hydrogel was formed in a 1 mL syringe, which was then swollen in PBS for 6 h in 48-well plates before the cell solution was added. Since the hydrogels did not occupy the whole bottom of each well, the cells were able to move freely off the hydrogel surface and settle at the bottom if they were not compatible with the surface. Figure 6B,C shows the adhesion and proliferation of Raw 264.7 cells on MoDPEG+ hydrogels with 1 wt % PP (Figure 6B) and pristine MoDPEG hydrogels (Figure 6C). Compared with pristine hydrogels, cells were more likely to spread on the MoDPEG+ hydrogel surface on day 1, indicated by smaller cell clusters and relatively even distribution on the MoDPEG+ hydrogel surface. Raw 264.7 cells on the pristine hydrogels formed much bigger clusters and multiple layers, suggesting that the cells did not adhere to the bio-inert PEG hydrogels. Interestingly, Raw 264.7 cells started to move away from the pristine MoDPEG hydrogel surfaces and there were few cells left on the hydrogel by day 5 (Figure 6C). The cells that moved away from the hydrogel surface formed bigger multiple layer cell clusters at the bottom of the plate on day 5 (Figure S3), which further indicated the bio-inert property of the pristine PEG hydrogels. On the other hand, Raw 264.7 cells showed good adhesion and obvious proliferation on MoDPEG+ hydrogel surfaces. There was a significant increase in cell numbers on day 5 (Figure 6B). For Hacat cells, the cells spread better on the MoDPEG+ hydrogels (Figure S4), but there was no visible proliferation over the timescale observed. With the MoDPEG hydrogels, the Hacat cells formed bigger, multiple layer cell clusters, in a similar fashion to the Raw 264.7 cells. Cell adhesion and proliferation were also investigated with hydrogels with 4 and 8 wt % PP, but due to the opaque nature of these gels, it was hard to observe cells and their behavior with an optical microscope. However, there were no obvious large cell clusters observed on either of these hydrogels (Figure S5). These results suggested that the incorporation of PP within the synthetic PEG hydrogels significantly increased the bioactivity of the hydrogels by allowing for the adhesion and proliferation of cells. The MoDPEG+ hydrogels, therefore, showed potential as skin tissue regenerative scaffolds.

### 3.5 Proof-of-Concept of Skin and Bone Regeneration Methodologies and 3D Printing

**Figure 7:**
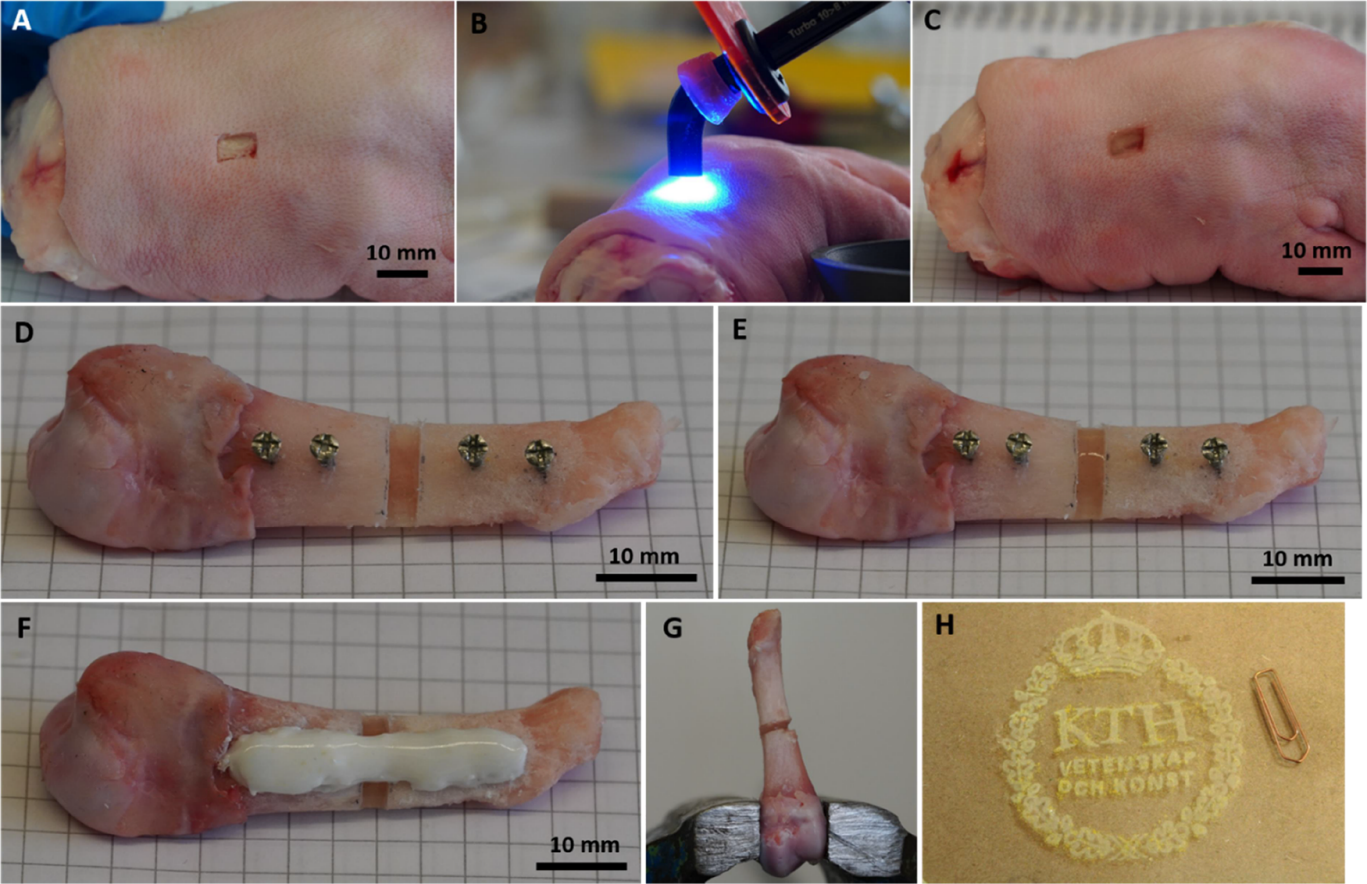
(A) Cuboid skin defect on porcine feet. (B) HEV curing of the MoDPEG+ precursor solution within the porcine skin wound using a dental lamp. (C) Skin wound filled with the cured MoDPEG+ hydrogel. (D) Unicortical bone void in a porcine metacarpal bone. (E) Cured MoDPEG+ hydrogel within the bone void. (F) Example of the MoDPEG+ hydrogel being used together with a screw and composite-based bone fixation system,^44^ which would support the bone fragments during healing. (G) Adhesive strength of the MoDPEG+ hydrogel allowed for the bonding of two porcine bone fragments. (H) Photograph of the swollen KTH logo printed with a SLA printer using the 6K50% MoDEPG+ hydrogel (8 wt % PP) precursor solution. Agglomerations of the PP and Tartazine can be seen as yellow spots in the print. The scale is indicated by the paper clip, which measured 32 mm.

The application of the MoDPEG+ hydrogel (10K20%) was demonstrated on a porcine skin wound and a bone void in a porcine metacarpal. The hydrogel solution was applied to the skin wound (8 × 5 mm × 1 mm, length × width × depth) by pipette (Figure 7A) and cured with 10 s of exposure to high-energy visible light (HEV) from a handheld dental curing lamp (Figure 7B). The surface tension of the hydrogel solution allowed the wound to be filled without run-off, while after curing the hydrogel adhered well to the porcine skin (Figure 7C). The hydrogel could also be used to fill a bone void (11 × 4 × 3 mm, depth × width × height) in a porcine metacarpal without run-off (Figure 7D,E). In the example shown in Figure 7F, the bone void was also stabilized with a screw and composite-based bone fixation system developed in our research group,^44^ which could be used to maintain alignment of a bone fracture while the MoDPEG+ hydrogel aids in bone healing. The adhesion of the cured hydrogel to bone was sufficient to prevent easy dislodgement of the hydrogel from the bone void. Moreover, the hydrogel’s adhesion was strong enough to hold two bone fragments together (Figure 7G). The adhesion to the porcine skin and bone substrates and the ease of the application and curing of the hydrogel within these wounds suggested that in situ application of the hydrogel should be feasible.

The 3D printability of the 6K50% MoDPEG+ hydrogel with 8 wt % PP was investigated with a SLA printer. The penetration depth for one layer of the hydrogel at an energy of 472 mJ·cm^–2^ was 6640 μm (Figure S6). This depth could be reduced to 100 μm by adding 0.4 wt % of the photoabsorber Tartrazine to the hydrogel formulation (Figure S7). The critical curing energy of the hydrogel was 79 mJ·cm^–2^. An example print was made of the KTH logo, which can be seen in Figure 7H. The high resolution and intricate details of the printed KTH logo demonstrated the potential for 3D printing these hydrogel systems even with high PP content. However, the placenta powder was not entirely stable in the hydrogel mixture during the printing process, which resulted in yellow accumulations of the powder and photoabsorber in the final print. Optimization of these systems for 3D printing will be done in our future work.

## 4 Conclusions

The successful decellularization and processing of human placenta made it possible to repurpose medical waste through incorporation of the processed tissue into synthetic PEG hydrogel scaffolds for tissue engineering. MoDPEG+ hydrogels were made using low-cost materials and facile fabrication procedures. The addition of placenta particles in the hydrogel formulation resulted in a material that was biocompatible, 3D printable and possessed tunable mechanical property through adjustments to the dry weight contents, PEG length and PP contents. In general, the addition of PP increased the mechanical strength and decreased the swelling ratios of the hydrogels. Critically, MoDPEG+ hydrogels mimicked natural tissue by presenting ECM biopolymers, and both Raw 264.7 and Hacat cells showed good adhesion to the MoDPEG+ hydrogels. Raw 264.7 cells also showed obvious proliferation on MoDPEG+ hydrogels. Additionally, MoDPEG+ could be easily applied to and cured within porcine skin and bone wounds, after which its adhesion to these substrates prevented the hydrogel from being dislodged.

3D printability of the hydrogels was demonstrated using a commercial SLA 3D printer, indicating that fabrication of PP-containing hydrogels with complex topology could be accomplished with continued efforts in fine-tuning the 3D printing parameters. With their highly tunable mechanical properties, good cytotoxicity profile and processability through 3D printing, the MoDPEG+ hydrogels presented in this study are promising candidates for fabricating precise tissue engineering scaffolds.

## Supporting Information Available

Experimental details; information about placenta donors; microscopic images of the hydrogels; swelling of the hydrogels; microscopic images of Raw 264.7 cells at the bottom of 48-well plate; microscopic images of Hacat cells on pristine and MoDPEG+ hydrogel; microscopic images of MoDPEG+ (10KPEG) hydrogels with PP content of 4 and 8 wt % after Raw 264.7 cell loading; a Jacob’s working curve and 3D printer *z*-axis resolution control by plotting laser penetration depth against photoabsorber, Tartrazine, concentration (PDF)

## Notes

Placentas were donated anonymously with informed consent from donors. The method of delivery, along with the age and ethnicity of the mother is included in the supporting information. With informed consent, approval of the research by an ethics committee was not necessary due to the anonymity of the donors.
