# Supplementary material for: Placenta Powder-Infused Thiol-Ene PEG Hydrogels as Potential Tissue Engineering Scaffolds
Source: Biomacromolecules. 2023 Mar 21;24(4):1617–26. doi: 10.1021/acs.biomac.2c01355 (PMC10091351; doi:10.1021/acs.biomac.2c01355)
Supplement: Supplementary file 1 — bm2c01355_si_001.pdf [file bm2c01355_si_001.pdf]

Supporting information:

Placenta Powder-Infused Thiol-Ene PEG Hydrogels as Potential Tissue Engineering Scaffolds

*Yanmiao Fan, Mads Lüchow, Adel Badria, Daniel J. Hutchinson, Michael Malkoch**

Division of Coating Technology, Department of Fibre and Polymer Technology, KTH Royal Institute of Technology, Teknikringen 56-58, 10044 Stockholm, Sweden

* Corresponding author: E-mail: malkoch@kth.se

Placentae were donated anonymously with informed consent from donors. The duration of the pregnancy and method of delivery, along with age and ethnicity of mother was recorded (**Table S1**). With informed consent, approval of the research by an ethics committee was not necessary due to the anonymity of the donors.

Table S1. Data on placentae and donor.

Delivery	Pregnancy duration (weeks + days)	Age of mother (years)	Ethnicity
**Cesarean section**	39 + 2	33	Caucasian
**Cesarean section**	37 + 2	37	African

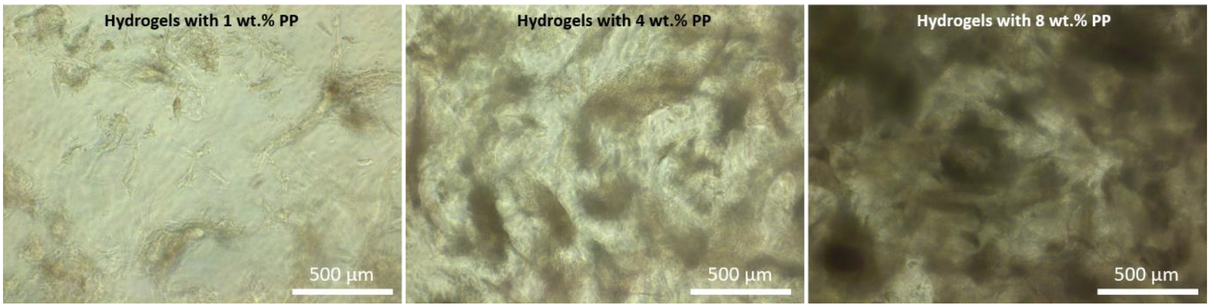

Figure S1. Microscopic images of MoDPEG+ (10KPEG) hydrogels with PP content of 1 wt.%, 4 wt.% and 8 wt.%.

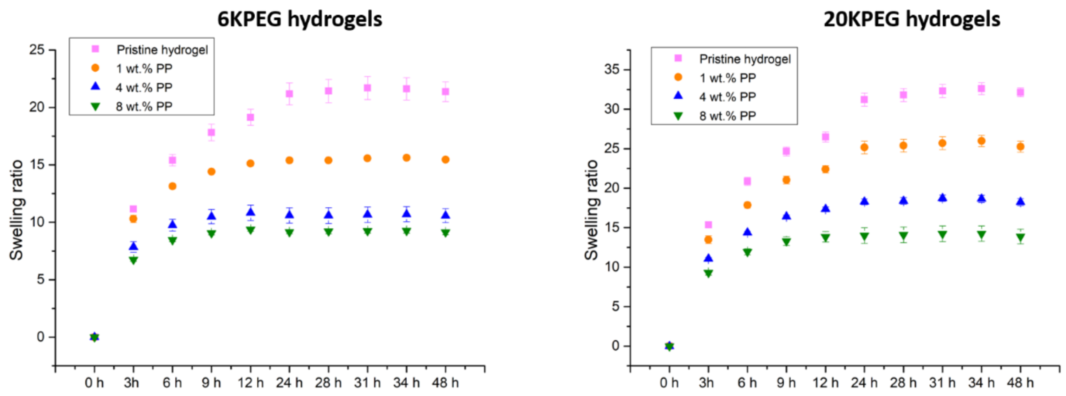

Figure S2. Swelling of the pristine (MoDPEG) and PP encapsulated (MoDPEG+) hydrogels.

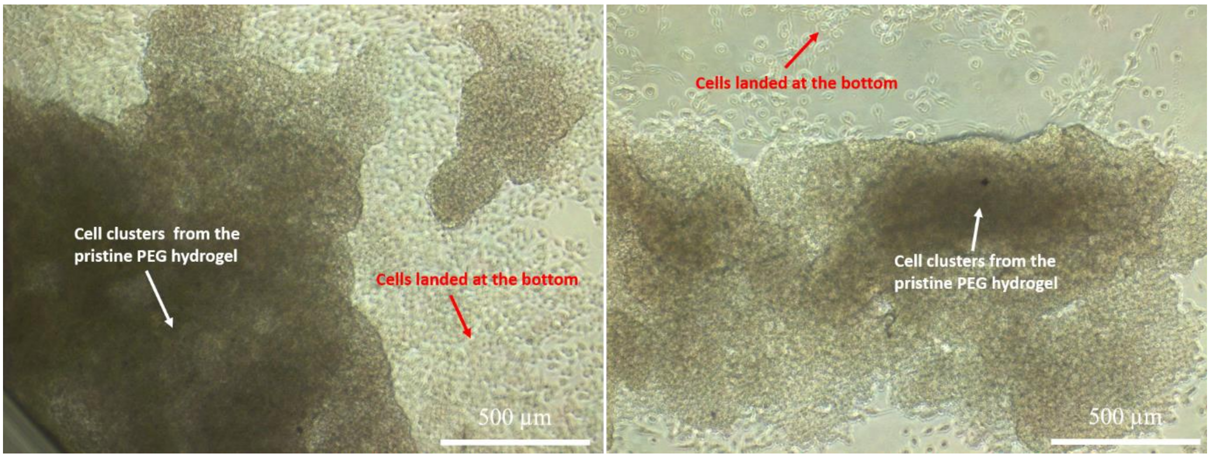

Figure S3. Microscopic images of the bottom of 48-well plate containing the pristine MoDPEG (10KPEG) hydrogel on day 5. The white arrows show Raw 264.7 cells moved away from the hydrogel surface, and the red arrows showed the cells landed at the bottom during cell loading procedure.

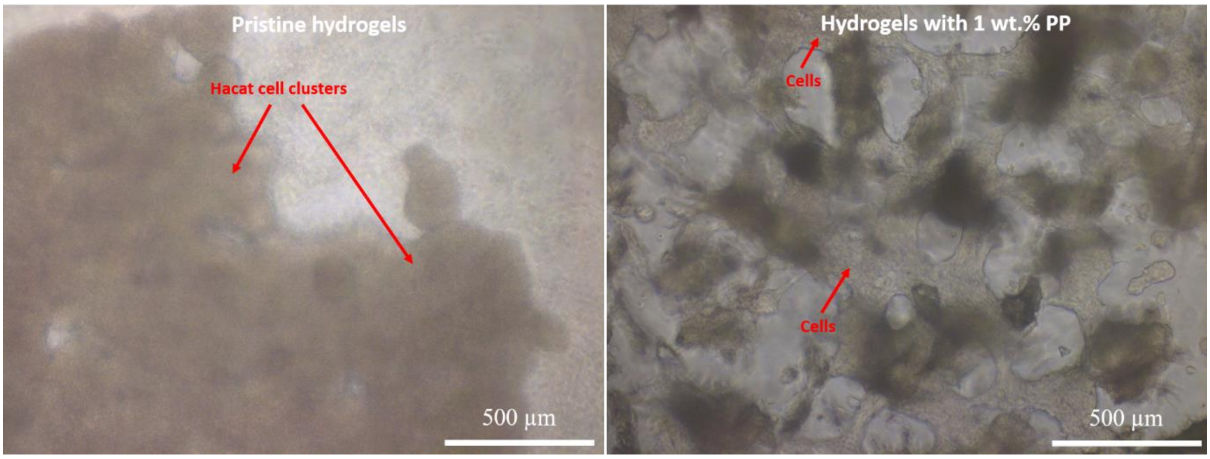

Figure S4. Microscopic images of Hacat cells on pristine and MoDPEG+ hydrogel (10KPEG, 1 wt.% PP).

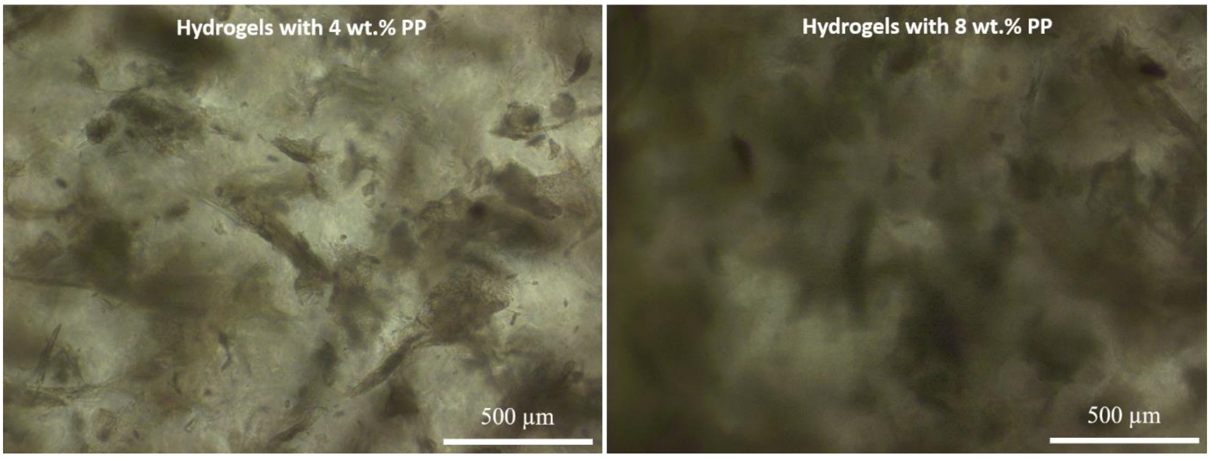

Figure S5. Microscopic images of MoDPEG+ (10KPEG) hydrogels with PP content of 4 wt.% and 8 wt.% after Raw 264.7 cell loading.

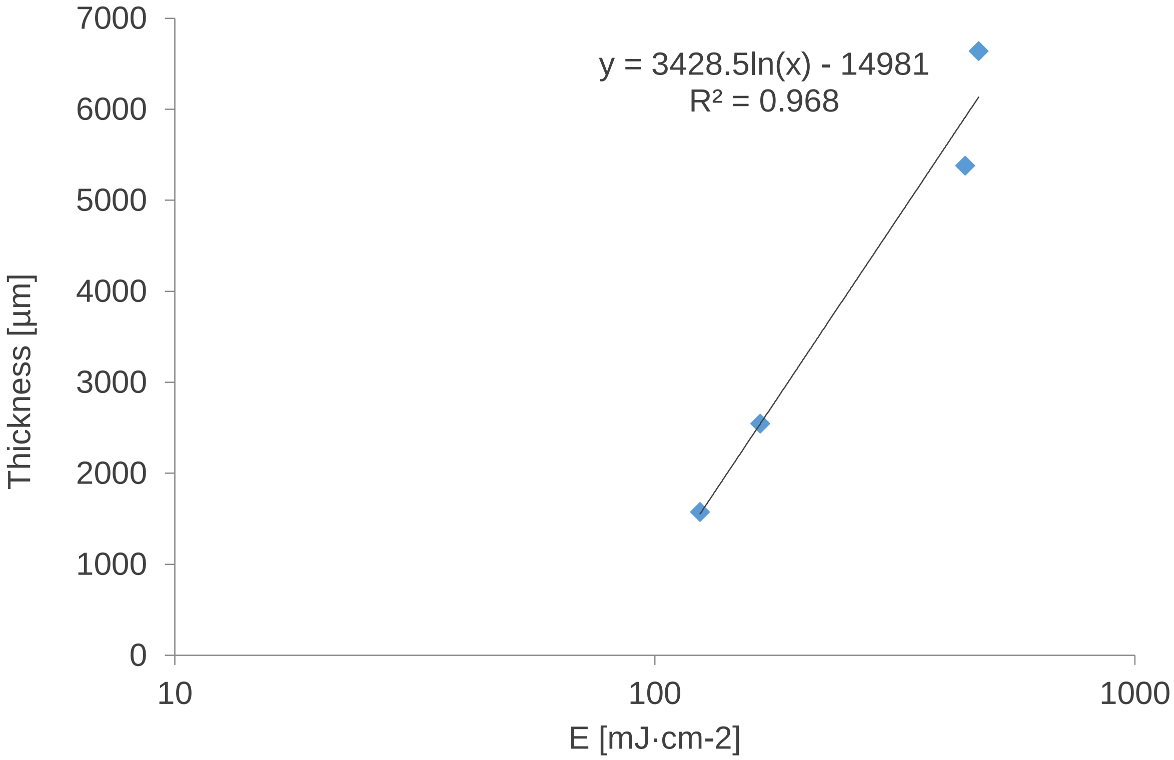

Figure S6. A Jacob's working curve produced by plotting curing thickness against laser energy, E. R^2 is related the function of the linear fit of the curve, y.

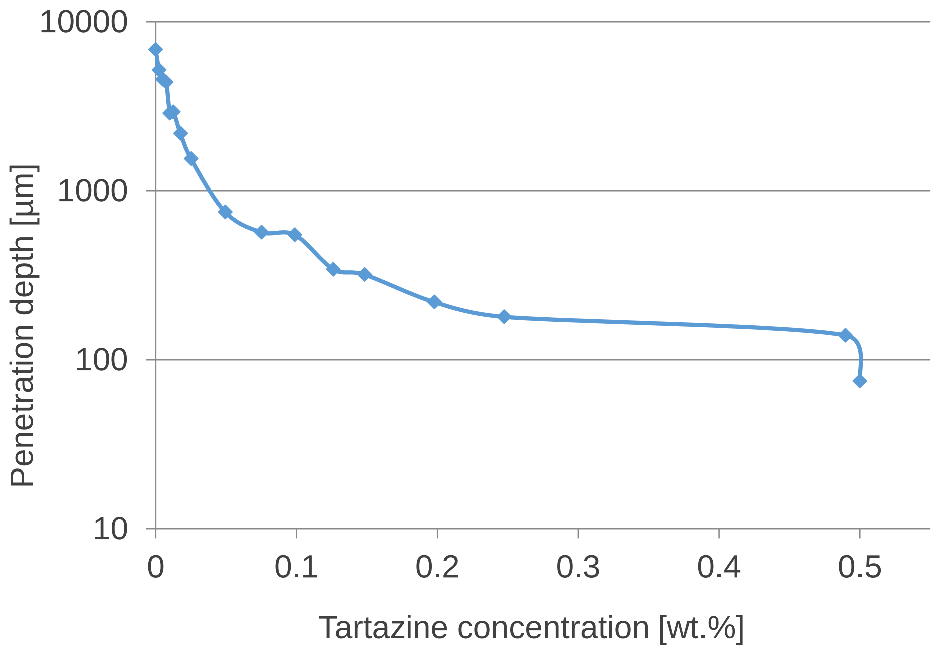

Figure S7. 3D printer z-axis resolution control was investigated by plotting laser penetration depth against photoabsorber, Tartrazine, concentration. Penetration depth is shown on a logarithmic scale. photoabsorber concentration.
